# External focus of attention enhances arm velocities during volleyball spike in young female players

**DOI:** 10.3389/fpsyg.2022.1041871

**Published:** 2023-01-05

**Authors:** Lukáš Slovák, Javad Sarvestan, Takehiro Iwatsuki, David Zahradník, William M. Land, Reza Abdollahipour

**Affiliations:** ^1^Human Motion Diagnostic Centre, University of Ostrava, Ostrava, Czechia; ^2^Department of Natural Sciences in Kinanthropology, Faculty of Physical Culture, Palacký University Olomouc, Olomouc, Czechia; ^3^Translational and Clinical Research Institute, Faculty of Medical Sciences, Newcastle University, Newcastle Upon Tyne, United Kingdom; ^4^Department of Kinesiology and Exercise Sciences, University of Hawaiʻi at Hilo, Hilo, HI, United States; ^5^Department of Kinesiology, College for Health, Community and Policy, The University of Texas at San Antonio, San Antonio, TX, United States

**Keywords:** focus of attention, volleyball, velocity, external focus of attention, volleyball spike

## Abstract

The aim of this study was to investigate the effect of different volleyball-specific attentional focus instructions on arm velocities of a volleyball spike in young female volleyball players using the Statistical Parametric Mapping method. Twelve young female volleyball players (13.6 ± 0.6 years old, 1.8 ± 0.8 years of experience in volleyball training) were asked to perform a volleyball spike in a standing position in three different attentional focus conditions including internal focus (IF, i.e., pull back your elbow prior to transfer momentum), external focus, (EF, i.e., imagine cracking a whip to transfer momentum), and control (CON, i.e., no-focus instruction). A Qualisys 3D motion capture-system was used to track reflective markers attached to the arm, forearm, and hand. Consequently, four phases of the volleyball spike including wind-up, cocking, acceleration, and follow-through were analyzed. A one-way repeated-measure ANOVA using one-dimensional statistical parametric mapping (SPM1d) showed that players achieved greater velocities in the hand (*p* < 0.01), forearm (*p* < 0.01), and arm (*p* < 0.01) using the EF instructions from the start of the wind-up phase to the acceleration phase. *Post-hoc* (SPM1d-*t*-tests-paired) analyses indicated significantly greater arm, forearm, and hand velocities during the EF condition, compared to CON (*p* < 0.01, *p* < 0.01, and *p* < 0.01 respectively) and IF (*p* < 0.01, *p* < 0.01, and *p* < 0.01 respectively) conditions. These findings suggest that EF instructions had an immediate impact on increasing volleyball spike velocity from the start of the wind-up phase to the acceleration phase prior to ball contact.

## Introduction

In volleyball, the spike is one of the most effective attacks, with the success rate of volleyball spikes directly linked to the match success rate (Valadés et al., 2016). To better understand the keys to an effective spike, several studies have investigated the underlying biomechanical mechanisms of the volleyball spike (Fuchs et al., 2019; Sarvestan et al., 2020). Results from these studies have revealed that spike velocities, within the entire upper arm mechanism, is a key element to achieving a successful volleyball spike (Valadés et al., 2016; Fuchs et al., 2019). Similar to other upper arm throwing actions, proximal-to-distal sequencing is an important aspect of achieving maximal linear velocities of the segment endpoint, such as hand velocity during the spike (Putnam, 1993).

Proximal-to-distal sequencing in upper arm throwing activities is characterized by an efficient and coordinated sequence of joint motions (generated by muscles), starting from the proximal segments (initiating the movements, e.g., arm) of the chain toward the more distal segments (concluding the movement, e.g., hand; Serrien et al., 2018; Fuchs et al., 2019). The main principle is that each successive segment peaks later and faster compared to the previous segment (Escamilla et al., 1998). That is to say, each segment builds off the acceleration of the previous one. Moreover, higher peak velocities reflect greater neuromuscular activity with greater force generation when the athletes efficiently employ the proximal-to-distal coordination pattern (Wang et al., 2018). Due to variable spatiotemporal conditions, volleyball players are required to rapidly react and execute the volleyball spike as fast as possible (Zwierko et al., 2010; Faity et al., 2022). In addition, the volleyball spike is a multidimensional action that involves four phases consisting of wind-up, cocking, acceleration, and follow-through (Reeser et al., 2010). Velocity at the end of the acceleration phase of the volleyball spike (ball-hitting moment) can be considered as the most important phase with regard to the final transfer of force to the ball. To this end, training that improves optimal biomechanical arm sequencing and velocity is crucial for enhancing volleyball spike performance.

One approach to improving movement performance and efficiency may be found in adopting an external focus of attention during volleyball spike execution. Essentially, an external focus (EF) of attention refers to attention directed toward the effects of one’s movement or movement goal (e.g., ball, ball trajectory, instrument, or target). Conversely, an internal focus (IF) of attention refers to attention directed toward one’s body movements while performing an action (e.g., movement of an arm or joint). Verbal instructions that promote an external focus of attention have been shown to be more effective than verbal instructions that induce an internal focus of attention. Such findings have been found across a variety of different motor tasks, regardless of age, (dis)ability, and level of expertise (Wulf, 2013; Chua et al., 2021). In particular, an EF has been found to be more effective than an IF for motor tasks where velocity plays a crucial role for optimizing outcome performance (An et al., 2013; Lohse et al., 2014; Halperin et al., 2017; Kershner et al., 2019). For example, An et al. (2013) investigated the golf swing and reported an increased X-factor stretch during the backswing, carry distance, and angular velocities of the pelvis, shoulder, and wrist when adopting an EF (e.g., push against the left side of the ground as you hit the ball) relative to an IF (e.g., transfer your weight to your left foot as you hit the ball) or control (e.g., no-focus instruction) group. In a study on dart-throwing (Lohse et al., 2014), results indicated that an EF relative to an IF of attention improved outcome performance and functional variability as reflected by increased variability in the angles and angular velocity of the shoulder, elbow, and wrist joints of the throwing arm. Investigating a punching task, Halperin et al. (2017) found that “focus on punching the pad as fast and as forcefully as possibly” (EF) compared to “focus on moving your arm as fast and as forcefully as possibly” (IF) increased velocity and punch impact. In a study on a countermovement jump, Kershner et al. (2019) reported an increased mean velocity and squat jump height when participants were asked to “concentrate on pushing away from the ground as explosively as possible” (i.e., EF) versus when they were required to “concentrate on extending your knees and hips as explosively as possible” (i.e., IF). As such, evidence suggests that an EF relative to an IF promotes increased angular velocities in motor tasks where velocity is a critical element for successful performance.

Even though the above-mentioned studies have provided insight regarding the influence of attentional focus on kinematic parameters (e.g., velocity and variability), these studies have tended to consider the spatiotemporal characteristics of the movement as a unified whole (e.g., average velocity; Davids et al., 2003). While this approach produces an overall picture of the advantages of EF relative to an IF, consideration of the moment-to-moment (time-series) movement sequence may provide a more comprehensive picture of the spatiotemporal changes over the course of the movement execution (Davids et al., 2003). In this respect, a method that analyzes time waveforms rather than movement coordination over single time points should be used for monitoring kinematic changes (Bańkosz and Winiarski, 2021) when considering the effects of attentional focus instructions. To this end, Statistical Parametric Mapping (SPM) provides in-depth information about point-by-point (time-series) movement sequences across the entire movement execution (Penny et al., 2011). Indeed, SPM analysis is used for determining the time to peak velocity of tracked trajectories to discern the spatial and temporal changes of the movement, representing movement efficiency (Faity et al., 2022). As such, using a SPM analysis may be particularly useful when attentional focus instructions relate to controlling the process of the movement over the course of movement execution.

Consequently, the aim of the current study was to investigate the effect of attentional focus instructions on the moment-by-moment changes in upper-limbs velocity during the volleyball spike using SPM. In considering upper-limb velocity, a key element was to understand the coordinated proximal-to-distal sequence (or synchronized movement timing) that provides capacities for greater spike velocities. Hence, using an SPM analysis could provide a better indicator of how various attentional focus instructions lead to temporal changes during spike performance. As such, we hypothesized that an EF relative to an IF or control condition would result in higher arm, forearm, and hand velocities from the start of the wind-up phase to the acceleration phase of the volleyball spike. In other words, we hypothesized that the coordination patterns of arm-to-forearm-to-hand velocities (e.g., sequence of velocity generation) would be higher in the period between movement initiation and ball-hitting moment of the volleyball spike while adopting an external focus relative to an internal focus and control condition.

## Materials and methods

### Participants

Twelve adolescent female volleyball players (age: 13.6 ± 0.6 years, height: 170.1 ± 5.8 cm, weight: 57.6 ± 6.1 kg) were recruited for this study. Previous studies on attentional focus using athletes have produced results with a large effect size (e.g., Bell and Hardy, 2009; Porter and Sims, 2013). As such, we assumed a large effect size when performing an *a priori* power analysis. An *a priori* power analysis with G*Power 3.1 indicated that 12 participants would be sufficient to identify significant differences between conditions in a within-participants design with a power (1−β) of 0.80, a large effect size ƒ of 0.4 (η_ρ_^2^ = 0.14), the number of measurements = 3, correlation among repeated measures = 0.5, nonsphericity correction e = 1, and an α level of.05 (Faul et al., 2007). Participants had 1.8 ± 0.8 years of volleyball experience, but did not have any specific training on performing the volleyball spike when the ball is fixed and not moving. Participants reported no history of musculoskeletal injuries (i.e., muscle, ligament, and tendon rupture, joint dislocation, and bone fracture) within the past 1 year. Participants were not aware of the specific aim of the study and their legal guardian signed the written informed consent prior to the data collection. *The ethical committee of the Faculty of Education, University of Ostrava, approved this study (Ethic code: 45/2021)*, which is in line with the 1964 Helsinki Declaration and its later amendments.

### Apparatus and task

Participants were asked to perform a maximal standing volleyball spike to a stationary ball hanging from the ceiling, similar to Fuchs et al. (2019). The height of the ball was standardized for each participant based on the height from the floor to the middle of the dominant hand when fully extended vertically. During the volleyball spike, participants had both feet in contact with the floor, while the feet position was standardized using tape pasted to the floor. Each volleyball spike was performed in the same direction. Measurement was performed under quiet conditions in the kinematics lab (*Human Diagnostic Centre*) at the University of Ostrava.

**Figure 1:**
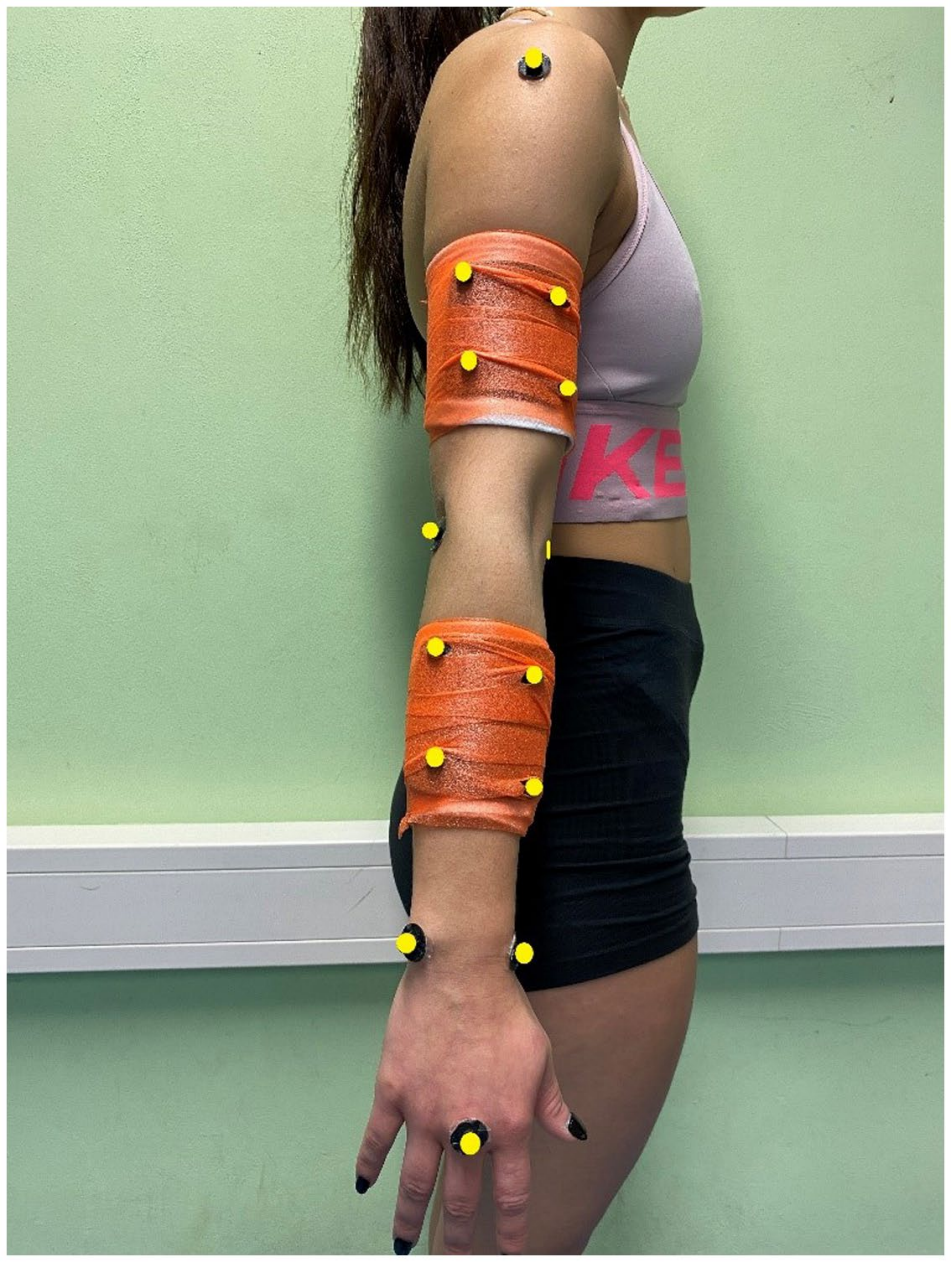
Marker placement on the participant dominant arm.

To track the kinematics of the volleyball spike, six 12 mm diameter markers and two clusters containing four markers were attached to the dominant upper limb landmarks (head of the third metacarpal, processus styloideus radii and ulnae, epicondylus lateralis and medialis humeri, and lateral part of shoulder) (C-motion, Rockville, MD, United States) (Figure 1). The data reconstruction and marker labeling were conducted using Qualisys Track Manager (Version 2021.1, Sweden) and Visual 3D software (C-Motion, Germantown, Kentucky, KY, United States). The interpolation method was used to fill the missing markers trajectories (not more than 10 frames). To identify the joints and segments, the corresponding static trial markers were used. A total of three segments were modeled (arm, forearm, and hand). Prior to data analysis, each spiking trial was trimmed from the start of the wind-up phase to the end of the follow-through phase, and was analyzed as a whole (Rokito et al., 1998; Sarvestan et al., 2020). Using the entire movement sequence, we identified four phases to allow for better illustration and interpretation of the outcomes and their application for training programs. The wind-up phase started with shoulder abduction and extension and ended with initiating the external shoulder rotation. The cocking phase started with shoulder external rotation and terminated with maximum shoulder external rotation. The acceleration phase started immediately after the cocking phase and finished when the upper arm was perpendicular to the trunk. The follow-through phase started with the arm perpendicular to the trunk and ended when the arm rotation was complete. Thereafter, the entire spike performance (trimmed data) was normalized to 100 data points for SPM analysis. Ten motion capture cameras (Oqus, Qualisys, Sweden) were used to record the spatiotemporal 3-dimensional trajectory including velocities of the attached marker and clusters with a sampling frequency of 240 Hz.

### Procedure

Prior to data collection, participants were instructed on the measurement process and instructed to focus solely on the assigned attentional instructions when performing the volleyball spike. Following a 10-min dynamic warm-up including stretching exercises and active joint mobility, passive reflective markers and clusters were attached to the dominant upper limb landmarks. Each player performed 3 practice trials of a standing volleyball spike with maximal force. Afterward, all participants performed 5 trials of the volleyball spike across three conditions (EF: external, IF: internal, and CON: control) with a one-min rest interval between the trials and a 3-min rest interval between focus conditions. The descriptive instructions for all participants were as follows: “*The task is to perform a volleyball spike with your dominant hand. The goal is to hit the ball as hard as possible*.” The descriptive instructions were provided to all participants to ensure an identical task goal (i.e., hitting the ball) across the different attentional focus conditions. Under the EF condition participants were further instructed to: “*Imagine cracking a whip to transfer momentum!*.” To ensure that participants understood the meaning of the external focus instructions, the experimenters asked participants whether they fully understood the meaning of cracking a whip. All participants reported their understanding of the given instructions. During the IF condition, participants were instructed: “*Pull back your elbow prior to transferring momentum!*” and finally, under the CON condition, no additional focus instructions were given. The order of the conditions was counterbalanced to eliminate the possibility of order effects. Attentional focus instructions were provided before each trial. Participants were not provided with performance feedback.

### Data analysis

Arm, forearm, and hand velocities were determined from the velocity of the respective modeled segment. Total segment velocity was determined using sum of vector velocities in anterior–posterior, mediolateral, and longitudinal axis and referenced to the lab space. The average moment-by-moment velocities from the normalized time series across the 5 trials for each condition were used for further statistical calculations. Ball velocity was not measured due to the limited space within the laboratory. Prior to data analysis, the Shapiro–Wilk normality test was employed to check the normality of the kinematic data (*p* > 0.05). One-way repeated-measures ANOVAs (SPM1d-ANOVA1RM for time-series analysis) were used to separately compare the arm, forearm, and hand velocities of the volleyball spike performance across the attentional focus conditions: EF, IF, and CON conditions (*α* < 0.05). Where inter-condition differences were highlighted, paired-sample *t*-tests (SPM1d-t-tests-paired, in time-series analysis) using Fisher’s least significant difference (since there were no more than three conditions) for *post-hoc* comparisons were performed (Hayter, 1986; Howell, 2010). For the entire analysis, we used the spm1d package (v0.4.3).^1^ The Partial Eta Square (η_p_^2^) values were calculated to interpret effect sizes. η_p_^2^ = 0.01 was considered as a small effect size, while η_p_^2^ = 0.06 and ≥ 0.14 were considered as moderate and large effect sizes, respectively (Sink and Mvududu, 2010). For the *t*-tests (*post-hoc*), the Cohen’s *d* ≤ 0.02 was considered as a small effect size, while the Cohen’s *d* ≤ 0.05 and ≥ 0.08 were considered as moderate and large effect sizes, respectively (Urdan, 2010). Since the SPM was performed for the entire volleyball spike performance, we were unable to report the exact effect size for each percent of the spike performance. We subsequently provided the effect size for each range (Sarvestan et al., 2021). All statistical analyses were conducted using MATLAB (v. 2021b, MathWorks, Inc., Natick, MA, United States).

## Results

**Figure 2:**
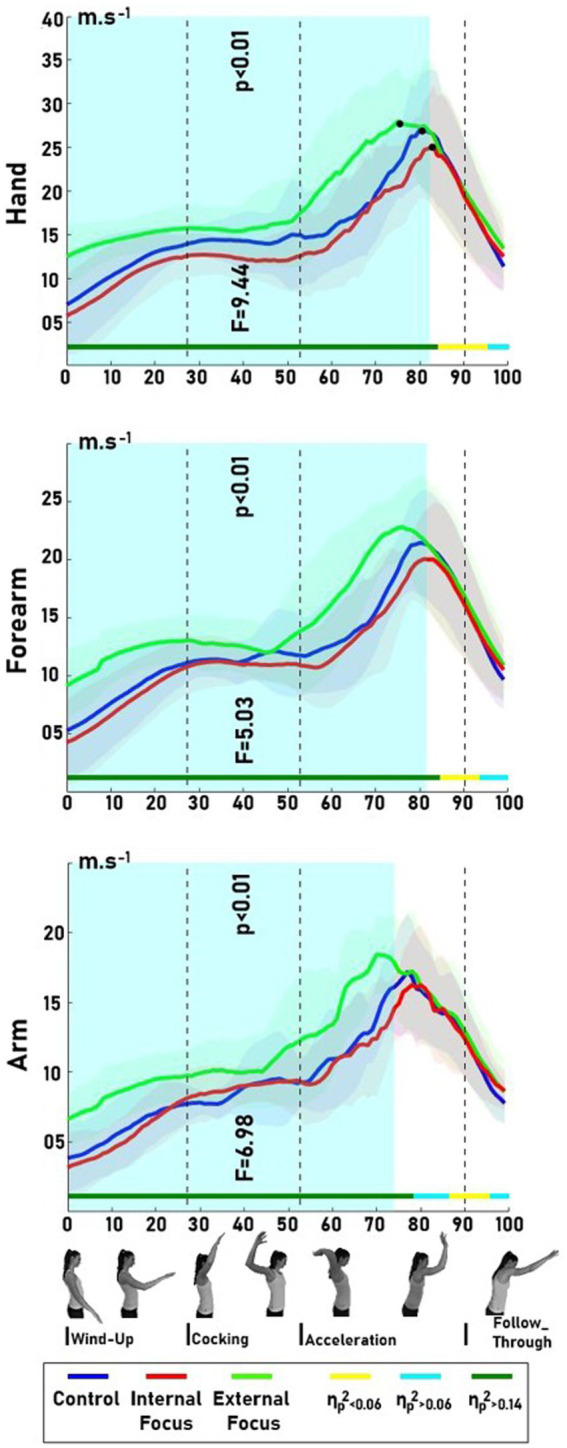
The arm, forearm, and hand velocities and their differences among players in three different spiking conditions: EF – external focus of attention (solid green line), IF – internal focus of attention (solid red line), and CON – control (solid blue line). The effect sizes were illustrated in dark green (η_p_^2^ > 0.14), cyan (η_p_^2^ > 0.06), and yellow (η_p_^2^ < 0.06) at the bottom of each graph. The black dots in hand velocities depict the approximate impact moment at each spiking condition. Identification of spike phases (i.e., cocking) is approximate as every player began each phase at a different time point.

A Shapiro–Wilk statistical test confirmed the normality of data distribution (*p* > 0.05). The SPM1d-ANOVA1RM depicted a significant difference among focus conditions in arm velocities beginning at movement initiation to 74% of the total movement, i.e., at the wind-up phase, cocking phase, acceleration phase, and after the ball-hitting moment (*F* = 6.98, *p* < 0.01, η_ρ_^2^ > 0.14) (Figure 2). *Post-hoc* analysis (SPM1d-t-tests-paired) showed a significantly greater arm velocity during the EF condition, compared to CON (0 to 75%, *p* < 0.01, *F* = 8.12, *d* ≥ 0.08) and IF (0 to 79%, *p* < 0.01, *F* = 8.81, *d* ≥ 0.08) conditions. No significant difference was observed between CON and IF conditions.

Similarly, in the forearm velocities, from the start of the wind-up phase to 82% (*p* < 0.01, *F* = 5.03, η_ρ_^2^ > 0.14) of the total movement, participants portrayed greater velocities in EF condition in comparison with CON (0–42 and 48–79%, *p* < 0.01, *F* = 7.53, *d* ≥ 0.08) and IF (0–83%, *p* < 0.01, *F* = 8.37, *d* ≥ 0.08) conditions. As for the hand velocities, participants reached significantly greater velocities from the start of the wind-up phase to 83% (*p* < 0.01, *F* = 9.44, η_ρ_^2^ > 0.14) of the total movement in the EF condition, compared to CON (0–77%, *p* < 0.01, *F* = 6.70, *d* ≥ 0.08) and IF (0–84%, *p* < 0.01, *F* = 9.72, *d* ≥ 0.08) conditions.

## Discussion

The present study contributes to the growing body of literature on the effects of attentional focus, and sought to extend this work by examining the spatiotemporal effects of attentional focus for enhancing upper arm segment velocities during volleyball spike performance. Specifically, the purpose of this study was to investigate the influence of different attentional focus instructions on upper-limbs velocities during a volleyball spike using SPM. Our findings support previous research highlighting the beneficial effect of an external focus of attention for motor performance (Wulf, 2013; Chua et al., 2021). Specifically, volleyball spike performance using an EF resulted in achieving significantly greater arm, hand, and forearm velocities than during CON and IF conditions. Moreover, the SPM analyses revealed that athletes reached peak arm, forearm, and hand velocities quicker (sharper curve slope) when performing with an EF. Such changes portray spatial and temporal efficiency within the movement (Faity et al., 2022), and reflect greater force generation (Wang et al., 2018).

The attentional focus instructions used in the current study were derived from biomechanical principles for enhancing arm velocities in volleyball. Specifically, the IF instruction: “*Pull back your elbow prior to transferring momentum!*” was an effort to enhance the stretch-shortening cycle (Dearing, 2018), and the EF instruction: “*Imagine cracking a whip to transfer momentum!*” was an effort to simulate correct proximal-to-distal sequencing of the upper limbs (Serrien et al., 2018). The hand velocity, as the most distal part of upper-limb kinematic sequence during ball-hitting or throwing activities, is considered the most crucial velocity for correlating with projectile (or ball) speed (Wagner et al., 2010, 2011; Lima et al., 2021). It is well known that optimal spiking and throwing techniques are performed by specifically ordered segment timing, including their acceleration and deceleration (Herring and Chapman, 1992; Wagner et al., 2011). Optimal joint movements occur in a proximal-to-distal order beginning with pelvis rotation, trunk rotation, and trunk flexion, followed by shoulder internal rotation, elbow flexion, and wrist and finger flexion (Wagner et al., 2012). Although the current study does not provide additional kinematics variables, (i.e., joint angles), we can suggest optimal progression of segmental motion under the EF instructions because the observed hand velocities reached their highest velocity compared to the other conditions (Serrien et al., 2018). Therefore, one can assume that the proximal-to-distal sequencing under EF was more effective at eliciting a whip-like motion (Herring and Chapman, 1992). That is, the initial segmental motion begins with the forward motion of a proximal segment while more distal segments rotate backward and then forward similar to whipping a whip. Additionally, we found significantly greater arm, forearm, and hand velocities under EF instruction which supports an overall positive effect of externally focused attention. Moreover, adopting the EF instruction in the current study demonstrated that athletes achieved greater arm, forearm, and hand velocity in a relatively shorter period (sharper curve slopes). This conveys greater segmental accelerations in the EF condition, which exhibits more force production by the muscular system (Maffiuletti et al., 2016).

The external focus instructions used in the current study (“imagine cracking a whip”) share similarities to instructions using analogies rather than EF instructions that direct attention toward movement effects, such as an implement, target, or a cue. Nonetheless, images and analogies have previously been used to induce an external focus of attention (Wulf et al., 1999, 2002; Lohse and Sherwood, 2011; Singh and Wulf, 2022). In this regard, studies have reported the benefits of an external relative to an internal focus by directing focus of attention to a pendulum-like motion of the club (external) as compared to focusing on the arm (internal) in a golf putting task (Wulf et al., 1999), focusing on an imaginary line (external) as compared to their thighs (internal) in a static wall-sit task (Lohse and Sherwood, 2011), or focusing on a “platform” (external) versus “arm” (internal) in a volleyball pass (Singh and Wulf, 2022). Likewise, Wulf et al. (2002) used images as attentional instructions observing that EF instructions (“hit the ball as if using a whip …”) were more effective than IF instructions (“snap your wrist while hitting the ball…”) when performing an overhand volleyball serve. Taken together, analogies have been shown to be effective to improve motor performance and learning (Lam et al., 2009a,b), and they could be used to produce the mental images of the movement goal which prevents the negative consequences of an internal focus (McKay et al., 2015; Singh and Wulf, 2022).

It is also important to consider that the IF condition did not demonstrate higher velocities in the forearm or hand at any point during the volleyball spike compared to the CON or EF conditions. This pattern of findings may reflect a disrupted and inharmonious movement pattern caused by the disruption of the automatic control processes resulting from the IF. To this effect, the participants could not properly and efficiently transfer the arm momentum to the distal segments (forearm and hand) and achieve greater spike velocities overall. Overall, participants adopting an internal focus produced similar results as the control condition in which no attentional focus instructions were given. This supports the notion that participants tended to spontaneously focus on their body movements (internal focus of attention), unless they are instructed otherwise (Land et al., 2013; Wulf, 2013). With regard to coaching, such findings reflect how even one or two different words in the attentional focus instruction can significantly change the motor outcome of the athletes (Wulf, 2013). As such, coaches and athletes should base pedagogical and training activities on the empirical results from research on attentional focus which would be beneficial for athletes’ performance and learning outcomes.

The current study highlights the benefit of using SPM to identify differences brought about by adopting an external focus of attention. Specifically, SPM was able to detect differences in the mobilization of peak velocities across the different attentional focus conditions. To this extent, findings indicated that an external focus facilitated more effective optimization (temporal pattern of peak velocities) of movement solutions compared to the internal focus or control condition. These findings have particular relevance for advancing theoretical insight and applied application of attentional focus findings. In particular, applying the SPM approach allows researchers to identify at what point within the movement the effect of an external focus impacts movement kinematics. Being able to more exactly identify how movements are being modified *via* adopting an EF has the potential to provide insight into the underlying mechanism of the EF advantage. Moreover, identifying the specific kinematic changes caused by adopting an EF could highlight potential critical elements within the movement, which would be important for guiding the focus of training. In the case of the current study, relatively decreased time to peak arm, forearm, and hand velocities may be an important element to consider during training. As such, more research is needed to explore the benefits and insights uncovered from applying SPM to understanding the advantages of adopting an external focus of attention.

Theoretically, the findings of this study could enrich our knowledge regarding the mechanisms underlying the advantages of an EF relative to an IF during the unfolding of the movement. Particularly, as the goal of the task in the current study was hitting the ball as hard as possible, the relatively earlier time to peak velocity might be an indicator of more effective and efficient coupling between the action and the goal. That is, participants in the EF condition reached the task goal more quickly than during the IF or control conditions, which could be an indicator of a more effective goal-action coupling (Wulf and Lewthwaite, 2016; Abdollahipour et al., 2017, 2022).

Essentially, an EF has been considered as a main contributor to goal-action coupling, which functions to enhance the linkage between the performer’s intended movement goal and the activation of one’s neuromuscular system (Wulf and Lewthwaite, 2016). Indirect evidence supporting the notion of EF enhancing goal-action coupling has been derived through observation of improved motor performance along with higher cognitive stability (as reflected by a lower number of eye blinks when adopting an EF; Abdollahipour et al., 2022). In another study on the relationship between attentional focus and inattentional blindness, children performed a bowling task while focusing on the path of the ball, their hands, or without focus instructions (Abdollahipour et al., 2017). Unbeknownst to the children, a 3-s video of individuals passing a basketball to each other was projected behind the target area when performing each bowling trial. In the final trial of each attentional focus condition, the same video was presented along with adding the well-known inattentional blindness stimulus showing a “gorilla” turning to face the camera, thumping its chest, and eventually turning away from the camera (Simons and Chabris, 1999). The findings showed that while performance outcome was superior in an EF relative to an IF and no-focus instruction conditions, children in the EF conditions noticed fewer distractive items than in IF and control conditions, indicating more concentration on the task goal. Taken together, the findings of the current study and previous studies (Abdollahipour et al., 2017, 2022) show that an EF may indeed promote an individual’s ability to focus on the task at hand or movement goal which suggests enhanced goal-action coupling (Wulf and Lewthwaite, 2016).

It is important to note, that the current study has several limitations. First, we did not provide information about ball speed and accuracy of the performance. Even though a very high correlation (*r* = 0.77) has been found between arm and ball velocities in volleyball spikes (e.g., Lima et al., 2021), it would still be interesting for future research to consider examining the effectiveness of attentional focus instructions on time to reaching peak velocity, ball velocity, and accuracy of performance outcome. Second, despite the fact that the participants were instructed to maximally adopt the attentional focus instructions, this study did not measure the extent to which participants adhered to the attentional focus instructions. Manipulation checks could be used in future research to estimate the amount of adherence to the instructions in internal and external focus conditions, as well as to determine what participants were focusing on during the control condition. Third, in the current study, participants performed the volleyball spike in a standing position in front of a stationary ball hanging from the ceiling. As such, the volleyball spike task could be made more ecological valid in future research. Fourth, it is recognized that in continuous data analysis, a larger sample size may be required (Robinson et al., 2021). Therefore, it is possible that the current study has only been sufficiently powered to detect effects slightly larger than those used in discrete parameter power analysis. Therefore, future research on the attentional focus that considers SPM analysis should use a larger sample size. Finally, while this study aimed to investigate the time-series of arm velocities, additional data analyses (e.g., angles, angular velocities) could provide further in-depth information regarding upper arm proximal-to-distal coordination. However, from a biomechanical point of view, arm velocities are crucial variables determining the spike success rate (Valadés et al., 2016; Fuchs et al., 2019).

## Conclusion

This study illustrates the importance that time-series analyses (i.e., SPM) can play in examining changes in motor performance under varied attentional foci. Specifically, a significant benefit was found from the start of the wind-up phase to the acceleration phase (prior to ball-hitting) under the external focus condition, when players produced greater arm, forearm, and hand velocities, compared to internal focus and control (no-focus instruction) conditions. That is, adopting an external focus of attention (“*Imagine cracking a whip to transfer momentum!*”) promoted fast, harmonized, and highly coordinated execution of the volleyball spike due to efficiently employing a proximal-to-distal coordination pattern. From a sports training perspective, the external focus instructions used in the current study may be highly applicable for volleyball players to increase velocities in the hitting arm. Lastly, future research should continue to use in-depth online time-series analyses to more precisely investigate the effects of attentional focus instructions on different movement tasks. Such analyses could result in both theoretical and applied insights.

## Data availability statement

The original contributions presented in the study are included in the article/supplementary material, further inquiries can be directed to the corresponding author.

## Ethics statement

The studies involving human participants were reviewed and approved by the Institutional Review Board of Faculty of Education, University of Ostrava, ethically approved this study (ethics code of 45/2021). Written informed consent to participate in this study was provided by the participants’ legal guardian/next of kin. Written informed consent was obtained from the minor(s)’ legal guardian/next of kin for the publication of any potentially identifiable images or data included in this article.

## Author contributions

All authors listed have made a substantial, direct, and intellectual contribution to the work and approved it for publication.

## Funding

The student grant competition of the University of Ostrava supported this study [2021/60]. Registration number: CZ.02.2.69/0.0/0.0/19_073/0016939.

## Conflict of interest

The authors declare that the research was conducted in the absence of any commercial or financial relationships that could be construed as a potential conflict of interest.

## Publisher’s note

All claims expressed in this article are solely those of the authors and do not necessarily represent those of their affiliated organizations, or those of the publisher, the editors and the reviewers. Any product that may be evaluated in this article, or claim that may be made by its manufacturer, is not guaranteed or endorsed by the publisher.

## Footnotes

^1^ www.spm1d.org
